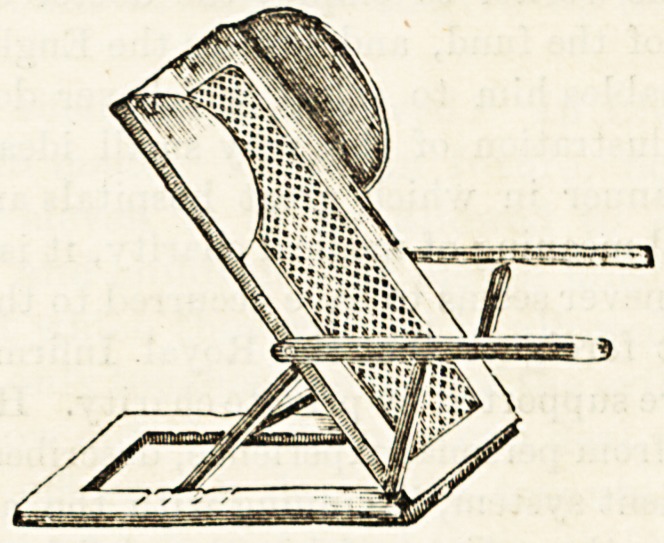# New Appliances and Things Medical

**Published:** 1899-10-07

**Authors:** 


### NEW APPLIANCES AND THINGS MEDICAL.

[We shall be glad to receive, at our Office, 28 & 29, Southampton Street, Strand, London, W.C., from the manufacturers, specimens of all new preparations and appliances which may be brought out from time to time.]

### THE "RASTON" NEW PATENT BED-RESTS.

(Messrs. Carter, 6a, New Cavendish Street, W.)

Bed-rests are always a trouble, and too often it is the case that, whatever else they may do, they fail to give rest. The origin of all the difficulty is that, in order to meet the requirements of different patients, and even of the same patient at different times, it is necessary that the bed-rest should be capable of being set at different angles, and that for this purpose it has generally been found necessary to have a bar across the bottom. In fact, the old style bed-rest consists essentially of a frame on to which the stuffing is fixed, attached by a pair of hinges to another frame lying on the bed, and raised and lowered by supports working in a rack on the lower frame. The hinges must be attached to the bar across the bottom edge of the frame, and, although this is out of the way when the patient sits very high, directly he is lowered the bar bores into his back and causes great discomfort. Messrs. Carter, however, in their new "Raston" bed-rest have got over this difficulty by replacing both the top and the bottom bars of the frame of the rest by bars of iron which, instead of lying in the same plane as the frame itself, are bent downwards out of the way of the patient's back (see illustration). By this simple device not only has this irksome bar been got rid of, but it has been found possible to replace the former somewhat rigid "ticking" which supported the cushion of the rest by an elastic and yielding woven wire network, like what is used for woven wire bedding. It may be asked, Why should not the old frame be fitted with the spring wire netting? But it was no use providing a yielding surface above so long as there was the rigid bar below. The more the patient's shoulders sunk into the one the more was the bottom of his back bored into by the other. But now the transverse bars both top and bottom are removed from the line of pressure, and the patient from the hips upwards is supported by the woven wire netting and the pillow or cushion fixed upon it. This is the patent, and there is no doubt it is a great improvement. The "Raston" is made in different sizes, some being complete bed-rests of the shape

Old Style.

The "Raston" as a Pillow Supp[illegible]rt.

New Style.

with which we are all familiar, supporting the head as well as the back, and some being made much narrower, so as rather to take the place of the bolster than to serve for patients who have to sit up. These are spoken of as pillow supports, and they seem likely to prove useful for people who can only sleep at certain angles and are awakened by the sinking of their pillows. Of course, a wire netting does not sink; moreover, the air space underneath keeps it cool.

### WELSBACH INCANDESCENT GAS LIGHT.

An exhibition of the Welsbach Incandescent Gas Light was given at the rooms of the company, York Street, Westminster, on September 29th. The decorative appearance of the light and fittings was exceedingly attractive, apart from all considerations of economy and efficiency. The Prince of Wales has just had Sandringham lighted by this company, and the elegance and efficiency secured by the recent improvements leave little, if anything, to be desired. A novelty designed to guard the mantles from accident caused by a knock is called the anti-vibrator. The whole burner is fitted on to a strong spring. This effectually breaks the force of any accidental blow, and so preserves the mantle. This spring is concealed by a more or less ornamental boss. Perhaps the improvement which will be most appreciated by the public is the disappearance of the tall chimney. The new burners require no chimneys, but a glass globe is recommended both as a protection against fire and as a support for an ornamental shade. In passing, it may be noted that pretty shades of fireproof silk are now made for the smaller lights, and thus one more danger from fire is practically obviated. The "Machebach" lighter is a very convenient addition, and possesses many advantages from a sanitary point of view. In the first place the old pilot light is done away with. The automatic lighter takes its place, and very fascinating it is to watch the gas light itself without visible agency. Its use is fully demonstrated in hospitals and public buildings, where the whole lighting can be controlled by one tap. A full description of the mechanism appeared in our columns last year. The "Kern" burner, of which so much has been heard of lately, is exceedingly ingenious, but it is nevertheless very simple. Gas and air, on the same principle as that of a Bunsen burner, are thoroughly mixed by their being passed through a long round burner, slightly constricted in the middle like a minute hollow lighthouse. The mingled gas and air then rush through a perforated cone, which regulates the pressure, into a round burner covered with the well-known incandescent mantle. A notched spreader prevents the gas lighting back into the cone. All burners can be fitted with automatic igniters. The principle of the "Kern" burner has been successfully adapted to oil lamps. The advantages claimed by this method of illumination are many. The quantity of gas and oil used is largely reduced—from 50 to 75 per cent. The light is stronger; the more perfect consumption reduces the by-products; thus a better and more wholesome light is secured at a large saving in cost. The fittings and igniter recommend themselves and excite great admiration wherever exhibited. The new inventions can in some cases be adapted to the old burners. Full particulars and prices can be obtained at any of the company's depôts.

### THYROGLANDIN (STANFORD).

(Evans, Son, and Co., Liverpool.)

The discovery of the powerful therapeutic action of the extract of the thyroid gland in cases of myxœdema, corpulency, and allied conditions was the starting point of what is now a popular and fashionable branch of medicine, namely, the special treatment of the disease of organs by variously prepared extracts of the corresponding healthy animal equivalent. Faith, engendered by the success of the thyroid treatment, has perhaps given an exaggerated value to many of these animal juices. About the thyroid treatment there can be no possible doubt. In the early days the freshly prepared juice of the sheep's thyroid was almost exclusively employed, and the results may fairly well be described as astonishing. The difficulty, however, of obtaining a regular supply of the glands, and the subsequent inconvenience and uncertainty of amateur methods, induced certain of the large manufacturing chemists to supply the prepared extract in fluid and desiccated forms. From this time forward failures were reported, and a certain want of confidence in the dosage began to creep into medical practice. Then a more or less successful attempt was made to separate the active principles, and supply them in standardised and constant strength. But the inherent difficulties of the process have rendered the results on the whole less uniformly successful than the original and perhaps crude method of preparing the extract from the fresh gland as required. Mr. Stanford's process, which has been exclusively adopted by Messrs. Evans and Son, appears to meet all objections; it affords a constant proportion of the two active principles of the gland, namely iodoglobulin and thyroiodin, and in the same proportion and in the same chemical condition as they exist in the fresh gland. Further than this, the product is sterilised according to a particular process, and will keep practically indefinitely without deterioration as regards therapeutic efficiency. Thyroglandin is supplied in the powder form, or, perhaps more conveniently, in pills. We believe that the medical profession are justified in using Evans' preparation with the greatest confidence, and anticipate that even better results than heretofore will be obtained by employing a preparation of such reliable and constant proportions.

### INVALID FURNITURE.

A visit to Messrs. Farmer, Lane, and Co.'s showrooms, 77 and 79 New Oxford Street, is evidence in itself of the great progress made of late years in the manufacture of furniture specially designed for the use of the sick. For one make of invalid couch or chair available a few years ago there are now dozens, and the choice is fairly bewildering. Of the newest designs mention should be made of this firm's New Combination Couch, an admirably constructed affair, ingeniously providing every kind of position and adjustment with lightness and portability. The three sections, back, middle, and leg, can be raised or lowered to any angle; the couch can be used as such, or as an armchair; the arms are movable, so that transit to and from bed is made easy for the cripple; the legs unscrew, and the whole frame can be folded to a comparatively small size for travelling. Made in birch and cane, this couch would be an admirable one for hospital ward use, and its price is moderate enough—£5. Of Improved Ilkley Couches, with their well-known advantages, Messrs. Farmer, Lane, and Co. have a good show; indeed, there seems no end to the contrivances employed for the greater comfort of those unhappily condemned to an invalid existence. We noticed some excellent bed-rests, light and firm, with a hinged back-piece which has manifest advantages. Carrying chairs of various makes, and some admirable hospital chairs, self-propelling and otherwise, of which many have been supplied to London hospitals, also deserve every commendation. Messrs. Farmer, Lane, and Co. are certainly to be congratulated, both upon their carefully-considered designs and the manner in which these are carried out in practical construction. The showrooms are well worth a visit.

## Figures and Tables

**Figure f1:**
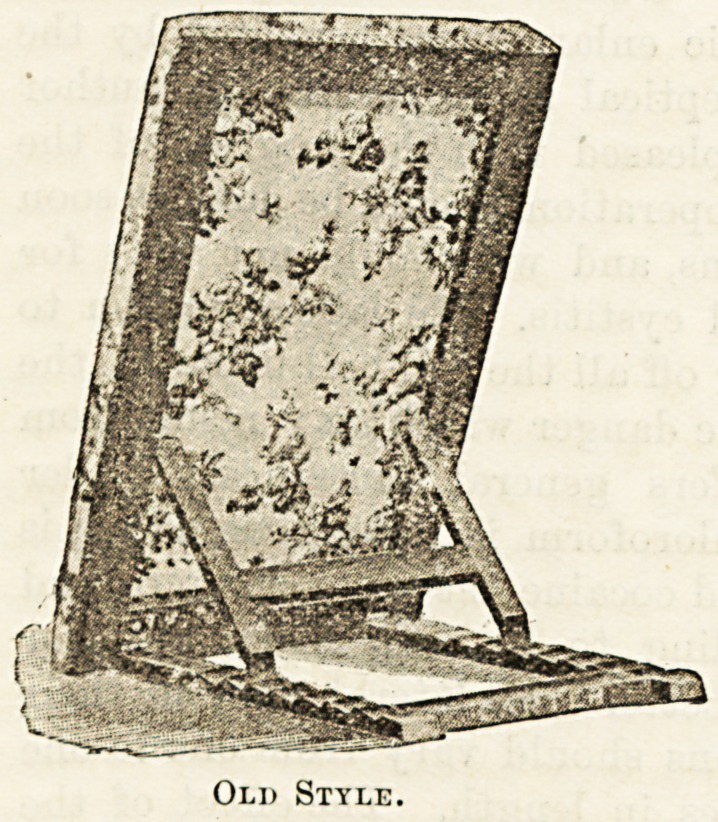


**Figure f2:**
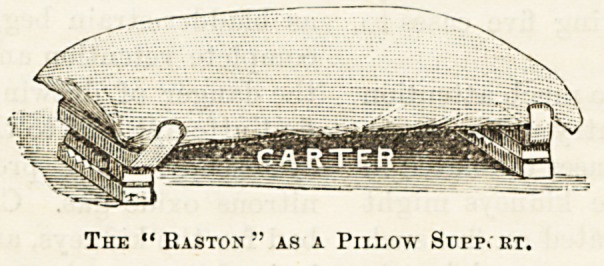


**Figure f3:**
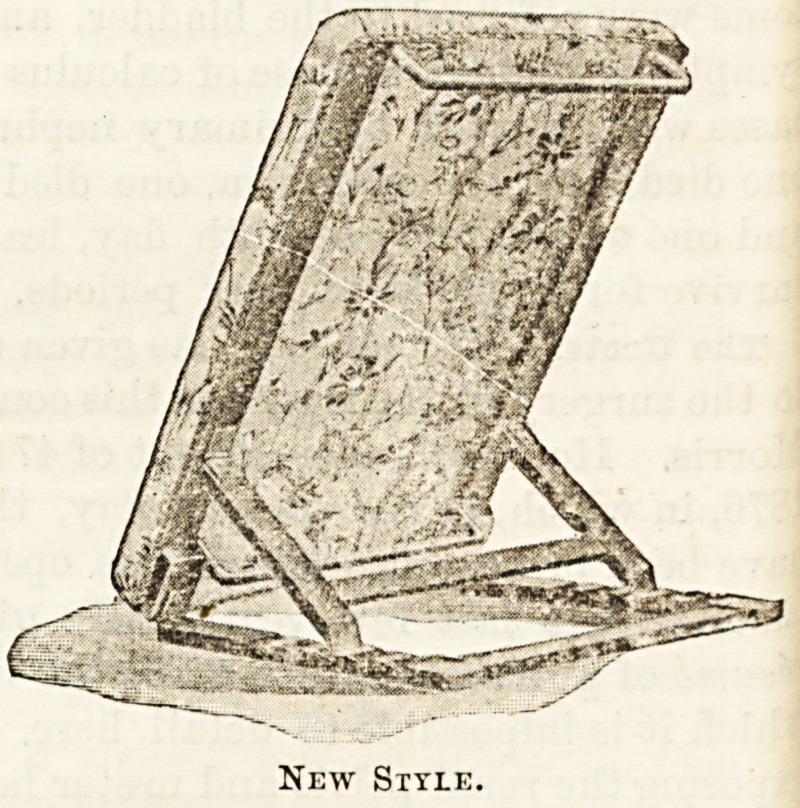


**Figure f4:**